# Relation between Condyle Horizontal Angle and Intercondylar Angle with Disc Displacement in Patients with Temporomandibular Joint Disorders: An MRI Evaluation

**DOI:** 10.1155/2023/3846525

**Published:** 2023-09-08

**Authors:** Shahriar Shahab, Zahra Amoozad Khalili, Elham Emami Meybodi, Morteza Banakar

**Affiliations:** ^1^Department of Oral and Maxillofacial Radiology, Faculty of Dentistry, Shahed University, Tehran, Iran; ^2^Department of Oral and Maxillofacial Medicine, School of Dentistry, Qom University of Medical Sciences, Qom, Iran; ^3^Department of Oral and Maxillofacial Radiology, Dental School of Shahid Sadoughi University of Medical Sciences, Yazd, Iran; ^4^Dental Research Center, Dentistry Research Institute, Tehran University of Medical Sciences, Tehran, Iran

## Abstract

**Background:**

Internal derangement (ID) is the most common cause of temporomandibular disorders (TMDs) and extensively affects the articular disc function. The anterior disc displacement is among the most important findings in ID. Knowledge about the etiology of this condition is imperative, and the role of structural parameters in the development of TMDs has not been well evaluated.

**Objectives:**

This study aimed to assess the relationship between condylar angulation and intercondylar angle with anterior disc displacement in patients with TMD using magnetic resonance imaging (MRI).

**Materials and Methods:**

This case-control study evaluated 31 temporomandibular joints with internal derangement and 57 normal joints. The data retrieved from MRI included disc position in the open mouth (normal, anterior disc displacement with a reduction (DDWR) and without reduction (DDWOR), and posterior displacement (PD)), horizontal condylar angle categorized as normal (10 to 30° angle) and abnormal (<10° and >30°), and intercondylar angle. Chi-square test, *T*-test, and Fisher's exact were done to assess the relationship between horizontal condylar angle and intercondylar angle in patients with TMDs with DDWR and DDWOR compared with the control group.

**Results:**

Patients with DDWR and DDWOR had higher odds of abnormal horizontal condylar angle, particularly >30° angle, which was a significant correlation (odds ratio of 0.19 and 8.3, respectively). The intercondylar angle in the patients with disc displacement was significantly smaller compared to the control group.

**Conclusion:**

Disc displacement was correlated with abnormal horizontal angle (particularly < 30) and smaller intercondylar angle compared with the control group.

## 1. Introduction

Management of temporomandibular disorders (TMDs) remains challenging for dentists [1]. The prevalence of TMDs is reported to range from 17% to 68% [2, 3]. The American Academy of Orofacial Pain (AAOP) defines TMD as a clinical condition that involves the temporomandibular joint (TMJ), masticatory muscles, and associated structures [2]. TMDs have various etiologies, such as internal derangement of the TMJ, disc abnormalities, dental problems, stress, trauma, and parafunctional habits [2, 4, 5]. Recent studies have also highlighted the potential role of hereditary factors in TMJ disc displacements. For instance, studies have reported associations between ESR1, COL5A1, and COL12A1 genes and the etiology of TMJ disc displacements [6, 7]. Furthermore, young female patients are more susceptible to developing TMDs [7]. The most common cause of TMDs is internal derangement, mainly related to anterior disc displacement [8]. It is missing to identify the exact etiologic factor that results in treatment failure and further disease progression [2, 4].

The TMJ structure and morphology might contribute to the development of TMDs. Studies have shown that changes in the joint structure produce clinical signs and symptoms in patients [4, 9]. Theoretically, changes in the joint structure can predispose patients to TMDs. Some recent magnetic resonance imaging (MRI) studies approved the hypothesis that a possible correlation exists between the increase in condylar horizontal angle with disc displacement and internal TMJ derangement; however, others found no significant correlation [10–12]. This study used MRI images to determine if the horizontal condylar and intercondylar angles are associated with disc displacement in patients with TMDs.

## 2. Materials and Methods

The study was approved by the ethical committee of the Shahed University of Medical Sciences (IR.SHAHED.REC.1398.085). This case-control study was done on 44 patients, i.e., 88 TMJs, including 31 joints with internal derangement of TMJ and 57 normal joints as the control group, selected out of those referring to two radiology clinics in 2019. The patient group was seeking treatment for TMD problems (pain, asymmetry, limitation of mouth opening, jaw deviation on opening, clicking, and joint sounds) [13]. They underwent MRI and were evaluated clinically to confirm the presence of the TMD sign and symptoms. The control subjects were selected from those who were taking brain MRI and were clinically examined prior to the MRI to ensure that they did not have any clinical signs and symptoms of TMD. Inclusion criteria were patients who underwent MRI and were evaluated clinically either to confirm the presence of TMD signs and symptoms or those who did not have any signs and symptoms of TMD. This study excluded those under 18 years of age, history of trauma/surgery, and unclear images. MRI images of both groups were obtained from two radiology clinics and evaluated by two skilled radiologists (Z.AK and E.EM). In cases where the two skilled radiologists disagreed in their evaluations, disagreements were resolved through consensus. If consensus could not be reached, a third evaluator (S.S) was consulted to provide an additional perspective and help reach a final decision.

**Figure 1:**
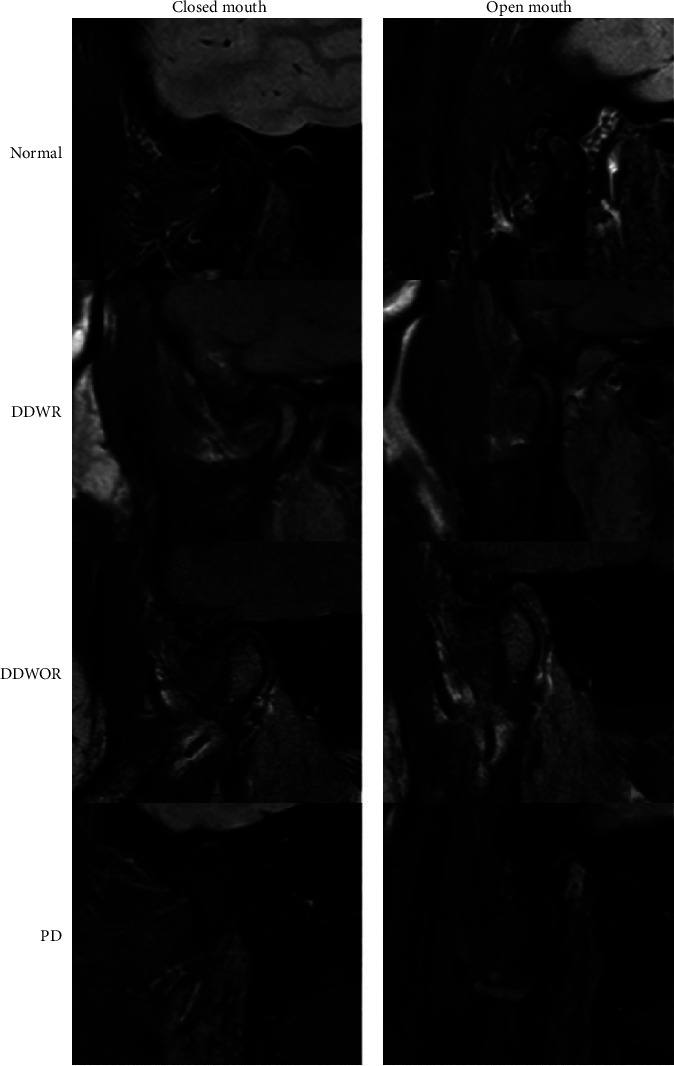
TMJ disc positions obtained from MRI of the closed-mouth and open-mouth positions showed normal DDWR, DDWOR, and PD. DDWR: disc displacement with reduction, DDWOR: disc displacement without reduction, and PD: posterior displacement.

For the open-mouth position, participants were instructed to open their mouths until they experienced no discomfort, and then their mouths were stabilized with a bite block. The articular disc position was determined using MRI images and categorized into the following groups (Figure 1) [14]:

1. *Normal*. In the closed-mouth position, the posterior band of the disc is located above the apex of the condyle. In the open-mouth position, the middle band of the disc is positioned between the condyle and the articular eminence.
2. *Anterior Disc Displacement with Reduction (DDWR)*. In the closed-mouth position, the posterior band of the disc is in front of the apex of the condylar head. In the open-mouth position, the disc-condyle relationship is similar to that of the normal position.
3. *Disc Displacement without Reduction (DDWOR)*. The posterior band of the disc is in front of the apex of the condylar head in both open- and closed-mouth positions.
4. *Posterior Displacement*. The disc is locked posteriorly in the closed-mouth position, resulting in an abnormal relationship with the condyle.

**Figure 2:**
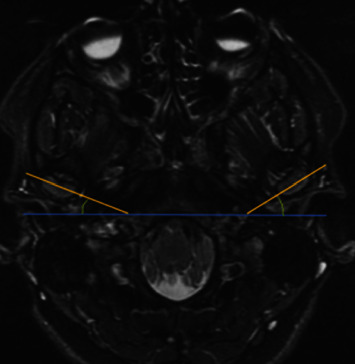
Horizontal condylar angle. The lateral and medial poles of the mandibular condyle in the closed-mouth position are connected, and a transverse line is drawn to connect the posterior region from right to left. The imaginary transverse line passes through the basion and is perpendicular to the midsagittal plane. The horizontal condylar angle is the angle between the transverse line and the line connecting the medial and lateral poles of the mandibular condyle.

**Figure 3:**
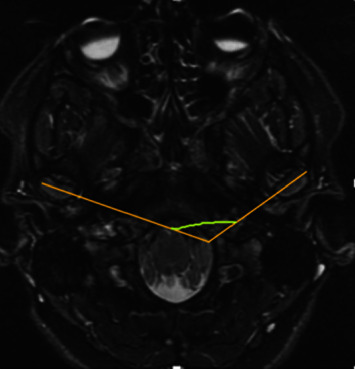
Intercondylar angle. The mediolateral axis of condyles is traced, and the angle between those two lines is the intercondylar angle.

Horizontal condylar angle or condylar angulation was measured as the angle in the axial plane between the intersection of a line connecting the two poles of the condyle and a hypothetical transverse line that passes through the basion and is perpendicular to the midsagittal plane. It was categorized as normal (10 to 30° angle) and abnormal (<10° and >30° angle) (Figure 2). The intercondylar angle was determined by marking and connecting the lateral and medial poles of the mandibular condyles and measuring the angle between the intersection of the two axes (Figure 3) [10, 13, 15].

MRI was done by using GE Signa Scanner (General Electric; Milwaukee, WI, USA) at 1.5 T, by using a TMJ surface coil with a 0.6 m diameter (Signa; GE Medical Systems) in closed-mouth position, in habitual occlusion, and then in open-mouth position (maximal opening). Sagittal proton density images were taken in open- and closed-mouth positions (TE28/2 ms and TR1500 ms), at a 14 × 14 cm field of view, 3 mm thickness, and 0.5 mm intersection gap. Sagittal T2 FSE bilateral images were obtained in a closed-mouth position (TR3100 ms and TE83/5 ms), at a 14 × 14 cm field of view, 3 mm thickness, and 0.5 mm intersection gap. Axial images were obtained in closed-mouth position (T2WI) with TR4120 ms and TE81/9 ms, a 24 × 24 cm field of view, 3 mm thickness, and 0.5 mm intersection gap. Measurements were obtained using the eFilm software (Merge eFilm, Milwaukee, WI, USA).

The correlation between the horizontal condylar angle and intercondylar angle in patients with TMDs and DDWR and DDWOR was analyzed and compared with the control using the chi-square test, *T*-test, and Fisher's exact test.

## 3. Results

This study evaluated 19 patients with TMDs (4 males and 15 females) and 25 controls (12 males and 13 females). The mean age was 35.42 ± 12.26 years in the case group and 40.52 ± 16.42 years in the control group.  Table 1 presents the frequency distribution of participants based on the kind of disc displacement (DDWR, DDWOR, PD, and DD) and horizontal condylar angle. Accordingly, the odds of abnormal horizontal angle were higher by 36% in DD and by 9% in the normal group (*P* < 0.001; odds ratio (OR): 1.50). Likewise, DDWR and DDWOR had higher odds of abnormal horizontal angle (OR: 0.19 and 8.37, respectively; *P* < 0.01), horizontal angles >30 being more common.

As displayed in Table 2, the mean intercondylar angle was 140 ± 12.3 in the control group and 132 ± 16.8 in the patient group, which was proved to be statistically significant based on the results of the *T*-test (*P* < 0.05). Concerning the obtained angles, the actual intercondylar angle meets the minimum requirements in both the control (confidence interval: 134–146) and the patients' group (confidence interval: 117–145).

## 4. Discussion

Our study showed that the disc displacement was correlated with abnormal horizontal angle (particularly < 30) and smaller intercondylar angle compared with the control group. The exact mechanism of the increase of horizontal angle in patients with advanced internal derangement is unknown. Advanced pathological changes in internal derangement and degenerative joint disorders are related to increased horizontal condylar angle due to forward and inward inclination [11, 16].

The supporting ligaments of TMJ adapt the articular disc between the osseous parts of the TMJ. This adaptation is affected by joint morphology, such as the condylar head inclination [17]. Kurita et al. [16] reported that increased horizontal condylar angle is associated with DDWOR and resorption of the condyle's lateral pole, which was supported by Westesson et al. [11]. Resorption of the lateral condyle can cause a change in the horizontal condylar angle. The resorptions are common in the middle and lateral 3^rd^ of the condyle, and bone formation, such as flattening and osteophyte formation, is more frequent in the anterior and superior area than in the posterior part. These changes justify the increased horizontal condylar angle. Apparently, due to the lateral attachment of the disc to the condyle, joints with a wider horizontal angle are prone to more stretching during anterior transitional movement. This lateral attachment is far less flexible than the posterior disc attachment; hence, overstretching would result in disc displacement and internal derangement of the TMJ [11, 16].

Inline with the present findings, similar MRI evaluations by Lee et al. [18] and Pregarz and Bodin [13] reported that increased horizontal angle was associated with disc displacement; abnormal inclination might effectively contribute to stomatognathic system dysfunction. De Stefano et al. [17] assessed the horizontal condylar inclination angle (between the sagittal midline in the axial plane) and the condyle's long axis and detected a significant relationship between the mean horizontal condylar angle and disc position, which was smaller when at rest, particularly in the DDWOR group.

Crusoé-Rebello et al. [19] reported that the anterior disc inclination in disc displacement disorders was associated with a horizontal angle >21°. Torres et al. [20] noted that both the increase and decrease of the horizontal angle could be associated with disc displacement. They attributed increased horizontal condylar angle to anterior-medial displacement and reduced angle to anterior-lateral displacement.

In a study by Sülün et al. [10], the DDWR and DDWOR were not different from the control group, yet the condylar angle in the control group was more symmetrical than that in the patient groups. They concluded a correlation between the horizontal condylar angle and internal derangement. The condyle in patients with internal derangement was flatter or tapered in the axial view, indicating that the symmetric or asymmetric wear of the anterior surface of the condyle would lead to degenerative changes or a remodeling process. Moreover, the excessive stretching of the lateral pterygoid muscle imposed a load on the condyle's lateral pole, consequently increasing the horizontal condylar angle. Sato et al. [21] and Amorim et al. [22] detected no significant relationship between the increased horizontal angle and condylar abnormality at an initial or advanced phase. However, Raustia and Pyhtinen [23] noted that the horizontal angle in patients with TMD was smaller than that in normal individuals.

Piancino et al. [24] found a significant relationship between the horizontal condylar angle difference on each side (asymmetric condyle) and the TMDs, being higher on average in patients with TMDs. The intercondylar angle and extent of disc displacement in the asymmetric cases were higher than those in the symmetrical group, but the difference was not significant. Based on the panoramic findings, increased vertical height in asymmetric cases was correlated with a higher risk of TMDs. The masticatory loading on TMJ causes the posterior inclination of the mandible and, subsequently, the anterior inclination of the condyle, which can ultimately result in TMDs. Changes in condylar angulation can cause bone remodeling following function and growth.

In the study by Eisenburger et al. [15], the mean intercondylar angle was measured to be 143° in the cases and 139° in healthy controls, which was not statistically significant. However, in the present study, the patients had smaller intercondylar angles compared with the control group.

It indicates an association between most functional disorders of the stomatognathic system and elevated activity of the lateral pterygoid muscle in bruxism [25, 26]. The inferior belly of this muscle has a complex attachment to the condylar neck, with variations in its insertions and origins reported in MRI studies [26–28]. High muscular tension can cause condylar deformity and rotation along the vertical axis. Accordingly, the intercondylar angle in patients with TMDs would be smaller than that in the rest of the population. Dysfunction in the joint and neuromuscular system might lead to intercondylar asymmetry. Oral habits might be detected through clinical examination, where changes in bone density of the contralateral condyle are noticed [15].

Our study was limited due to the small sample size and the absence of other imaging modalities, such as CT and MRI. However, its strengths were the use of magnetic resonance images, which displayed the exact disc position, as well as employing two skilled radiologists and considering a control group. Further studies are needed with a larger sample size for simultaneous clinical and radiological assessment and comparison of TMDs cases and controls in longitudinal studies. Comparisons can also be made between MRI and other imaging modalities.

## 5. Conclusion

This study showed that disc displacement with or without reduction is associated with abnormal horizontal angle (especially > 30°) and smaller intercondylar angle compared with the control group. Nowadays, many patients with TMDs are imaged by CBCT and/or MDCT (multidetector computed tomography), and accurate determination of condyle horizontal angle is possible with these techniques. It is recommended that TMD patients with increased horizontal angles and smaller intercondylar angles be considered to treat disc displacement. Early detection of TMDs with disc displacement enables clinicians to offer effective treatment more rapidly and at a lower cost and prevent further damage to delicate TMJ hard and soft tissue components.

## Figures and Tables

**Table 1:** Details of the participants based on the type of disc displacement and condylar angulation.

Variable	Category	DDWR	DDWOR	PD	Total	Normal
		9 joints	20 joints	2 joints	31 joints	57 joints
Horizontal condylar angle	Normal (10–30°)	7 (77.8%)	11 (55%)	2 (100%)	20 (64.5%)	52 (91.2%)
	Abnormal (<10, >30)	2 (22.2%)	9 (45%)	0 (0%)	11 (35.5%)	5 (8.8%)

**Table 2:** Intercondylar angle.

Groups	Mean ± SD	95% confidence interval	*p* value
Control (25)	12.3 ± 140	134–146	*P* < 0.05
Patient (19)	16.8 ± 132	117–145	

## Data Availability

The data used to support the findings of this study are available from the corresponding author upon request.

## References

[1] Ogura I., Kaneda T., Mori S., Sakayanagi M., Kato M. (2012). Magnetic resonance characteristics of temporomandibular joint disc displacement in elderly patients. *Dentomaxillofacial Radiology*.

[2] Chalkoo A. H., Bhat Z. A., Maqbool A. (2017). MRI characteristics of disc displacement of temporomandibular disorders and its correlation with clinical findings in symptomatic and asymptomatic. *Population*.

[3] Rokaya D., Suttagul K., Joshi S., Bhattarai B. P., Shah P. K., Dixit S. (2018). An epidemiological study on the prevalence of temporomandibular disorder and associated history and problems in Nepalese subjects. *Journal of Dental Anesthesia and Pain Medicine*.

[4] Murphy M. K., MacBarb R. F., Wong M. E., Athanasiou K. A. (2013). Temporomandibular disorders: a review of etiology, clinical management, and tissue engineering strategies. *The International Journal of Oral & Maxillofacial Implants*.

[5] Basat S. O., Surmeli M., Demirel O., Ceran F., Saydam F. A., Basaran K. (2016). Assessment of the relationship between clinicophysiologic and magnetic resonance imaging findings of the temporomandibular disorder patients. *Journal of Craniofacial Surgery*.

[6] Dalewski B., Białkowska K., Pałka Ł., Jakubowska A., Kiczmer P., Sobolewska E. (2021). COL5A1 RS12722 is associated with temporomandibular joint anterior disc displacement without reduction in polish caucasians. *Cells*.

[7] Dalewski B., Kaczmarek K., Jakubowska A., Szczuchniak K., Pałka Ł., Sobolewska E. (2021). COL12A1 single nucleotide polymorphisms rs240736 and rs970547 are not associated with temporomandibular joint disc displacement without reduction. *Genes*.

[8] Hasan N. M. A., Abdelrahman T. E. F. (2014). MRI evaluation of TMJ internal derangement: degree of anterior disc displacement correlated with other TMJ soft tissue and osseous abnormalities. *The Egyptian Journal of Radiology and Nuclear Medicine*.

[9] Hirata F. H., Guimarães A. S., Oliveira J. X. D., Moreira C. R., Ferreira E. T. T., Cavalcanti M. G. P. (2007). Evaluation of TMJ articular eminence morphology and disc patterns in patients with disc displacement in MRI. *Brazilian Oral Research*.

[10] Sülün T., Akkayan B., Duc J.-M. P., Rammelsberg P., Tuncer N., Gernet W. (2001). Axial condyle morphology and horizontal condylar angle in patients with internal derangement compared to asymptomatic volunteers. *CRANIO®*.

[11] Westesson P.-L., Bifano J. A., Tallents R. H., Hatala M. P. (1991). Increased horizontal angle of the mandibular condyle in abnormal temporomandibular joints: a magnetic resonance imaging study. *Oral Surgery, Oral Medicine, Oral Pathology*.

[12] Matsubara R., Yanagi Y., Oki K. (2018). Assessment of MRI findings and clinical symptoms in patients with temporomandibular joint disorders. *Dentomaxillofacial Radiology*.

[13] Pregarz M., Bodin C. (2010). *MRI Evaluation of TMJ Condylar Angulations*.

[14] De Farias J., Melo S., Bento P., Oliveira L., Campos P., De Melo D. (2015). Correlation between temporomandibular joint morphology and disc displacement by MRI. *Dentomaxillofacial Radiology*.

[15] Eisenburger M., Haubitz B., Schmelzeisen R., Wolter S., Tschernitschek H. (1999). The human mandibular intercondylar angle measured by computed tomography. *Archives of Oral Biology*.

[16] Kurita H., Ohtsuka A., Kobayashi H., Kurashina K. (2003). Relationship between increased horizontal condylar angle and resorption of the posterosuperior region of the lateral pole of the mandibular condyle in temporomandibular joint internal derangement. *Dentomaxillofacial Radiology*.

[17] De Stefano A. A., Guercio‐Monaco E., Hernández‐Andara A., Galluccio G. (2020). Association between temporomandibular joint disc position evaluated by magnetic resonance imaging and mandibular condyle inclination evaluated by computed tomography. *Journal of Oral Rehabilitation*.

[18] Lee P. P., Stanton A. R., Hollender L. G. (2017). Greater mandibular horizontal condylar angle is associated with temporomandibular joint osteoarthritis. *Oral surgery, oral medicine, oral pathology and oral radiology*.

[19] Crusoé-Rebello I. M. R., Campos P. S. F., Rubira I. R. F., Panella J., Mendes C. M. C. (2003). Evaluation of the relation between the horizontal condylar angle and the internal derangement of the TMJ-a magnetic resonance imaging study. *Pesquisa Odontológica Brasileira*.

[20] Torres M. G. G., Crusoé-Rebello I. M., Rosário M., Albuquerque M. C., Campos P. S. F. (2016). Morphometric features of the mandibular condyle and association with disk abnormalities. *Oral surgery, oral medicine, oral pathology and oral radiology*.

[21] Sato H., Fujii T., Kitamori H. (1997). The clinical significance of the horizontal condylar angle in patients with temporomandibular disorders. *CRANIO®*.

[22] Amorim M. Y., Alves M. G., Almeida J. D., Montesinos G. A., Costa A. L., Lopes S. L. P. D. C. (2019). Inclination of the condylar long axis is not related to temporomandibular disc displacement. *Journal of investigative and clinical dentistry*.

[23] Raustia A. M., Pyhtinen J. (1990). Morphology of the condyles and mandibular fossa as seen by computed tomography. *The Journal of Prosthetic Dentistry*.

[24] Piancino M. G., Tepedino M., Cavarra F. (2018). Condylar long axis and articular eminence in MRI in patients with temporomandibular disorders. *Cranio®*.

[25] d’Ippolito S., Borri Wolosker A., D’Ippolito G., Herbert de Souza B., Fenyo-Pereira M. (2010). Evaluation of the lateral pterygoid muscle using magnetic resonance imaging. *Dentomaxillofacial Radiology*.

[26] Finden S., Enochs W., Rao V. (2007). Pathologic changes of the lateral pterygoid muscle in patients with derangement of the temporomandibular joint disk: objective measures at MR imaging. *American Journal of Neuroradiology*.

[27] Ngamsom S., Nakamura S., Sakamoto J., Kotaki S., Tetsumura A., Kurabayashi T. (2017). The intravoxel incoherent motion MRI of lateral pterygoid muscle: a quantitative analysis in patients with temporomandibular joint disorders. *Dentomaxillofacial Radiology*.

[28] Wang S., Chen Y., She D. (2022). Evaluation of lateral pterygoid muscle in patients with temporomandibular joint anterior disk displacement using T1-weighted Dixon sequence: a retrospective study. *BMC Musculoskeletal Disorders*.

